# Implementing a tobacco-free workplace program at a substance use treatment center: a case study

**DOI:** 10.1186/s12913-024-10629-5

**Published:** 2024-02-14

**Authors:** Anastasia Rogova, Isabel Martinez Leal, Maggie Britton, Tzuan A. Chen, Lisa M. Lowenstein, Bryce Kyburz, Kathleen Casey, Kim Skeene, Teresa Williams, Lorraine R. Reitzel

**Affiliations:** 1 The University of Texas MD Anderson Cancer Center, 1400 Pressler Street, 77230-1402 Houston, TX Unit 1444, P.O. Box 301402, United States of America; 2 University of Houston, 3657 Cullen Blvd, Stephen Power Farish Hall, 77204 Houston, TX United States of America; 3 Integral Care, 1430 Collier St, 78704 Austin, TX United States of America

**Keywords:** Tobacco cessation, Tobacco-free policy, Health disparities

## Abstract

**Background:**

People with substance use disorders smoke cigarettes at much higher rates than the general population in the United States and are disproportionately affected by tobacco-related diseases. Many substance use treatment centers do not provide evidence-based tobacco cessation treatment or maintain comprehensive tobacco-free workplace policies. The goal of the current work is to identify barriers and facilitators to a successful and sustainable implementation of a tobacco-free workplace program, which includes a comprehensive tobacco-free policy and evidence-based cessation treatment services, in a substance use treatment center.

**Methods:**

This study is based on an ethnographic approach and uses a qualitative case study design. Data were collected via interviews with staff (*n* = 6) and clients (*n* = 16) at the substance use treatment center and site visits (*n* = 8). Data were analyzed using thematic analysis guided by the extended Normalization Process Theory designed to inform the implementation of innovations in healthcare practice.

**Results:**

Staff at the substance use treatment center supported the implementation of the program and shared a good understanding of the purpose of the intervention and its potential benefits. However, the study identified significant challenges faced by the center during implementation, including widespread tobacco use among clients, contributing to attitudes among staff that tobacco cessation was a low-priority problem due to a perceived lack of interest in quitting and inability to quit among their clients. We identified several factors that contributed to changing this attitude, including provision of tobacco training to staff, active leadership support, low number of staff members who smoked, and access to material resources, including nicotine replacement products. The implementation and active enforcement of a comprehensive tobacco-free workplace program contributed to a gradual change in attitudes and improved the provision of evidence-based tobacco cessation care at the substance use treatment center.

**Conclusions:**

Substance use treatment centers can integrate tobacco cessation practices in their daily operations, despite multiple challenges they face due to the complex behavioral health and socioeconomic needs of their clients. With proper support, substance use treatment centers can provide much needed tobacco cessation care to their clients who are disproportionately affected by tobacco-related health conditions and systemic health inequities.

**Supplementary Information:**

The online version contains supplementary material available at 10.1186/s12913-024-10629-5.

## Background

According to data from the Centers for Disease Control and Prevention, 12.5% of the US adults aged ≥ 18 years reported current use of cigarettes in 2020 [1]. While this figure represents a substantial decrease from over 40% of the adult population smoking in the 1960s, tobacco use is still the leading preventable cause of death in the US [2] with annual deaths directly attributable to tobacco use estimated to be at least 480,000 [3]. However, these devastating effects of tobacco use do not equally impact all population groups. The proportion of people who use tobacco products is dramatically elevated among the often intersecting groups of people experiencing socioeconomic disadvantage, who are medically underserved, and/or people living with comorbid mental health and non-nicotine substance use disorders [4]. These health disparity populations have disproportionately high smoking rates; for example, over 65% of adults with substance use disorders (and up to 90% according to some sources) are active smokers [5–7]. As a result, adults with substance use disorders are disproportionately affected by tobacco-related disease compared to the general population [7, 8]. 

There is an overwhelming body of evidence that adults with substance use disorders are interested in and capable of quitting with appropriate support [9, 10]. Current clinical guidelines recommend that all clients be provided with evidence-based cessation care, which includes behavioral interventions such as tobacco use assessment, brief cessation advice, individual or group counseling, and pharmacotherapy such as nicotine replacement therapy or non-nicotine medication (bupropion and varenicline) [11, 12]. Moreover, the adoption of system-level policies, including comprehensive tobacco-free workplace policies, which prohibit the use of any form of tobacco inside buildings and on the grounds of behavioral health treatment centers, are also shown to be effective in improving quit rates [13]. Despite their proven effectiveness, however, evidence-based practices and policies remain underutilized, and tobacco use treatment is given a low priority in substance use treatment centers. For example, according to a 2016 nationwide study, only 64.0% of substance use treatment centers reported screening clients for tobacco use, 47.4% offered tobacco cessation counseling, 26.2% offered nicotine replacement therapy, and 34.5% had tobacco-free policies [4]. Furthermore, although not reported, it is possible that some proportion of these centers had tobacco-free workplace policies that may have been non-comprehensive in product coverage (e.g., not extending to e-cigarettes/vaping) or workplace area coverage (e.g., allowing smoking areas), which are known to be less effective than their comprehensive policy counterparts [14–16]. Consequently, there is a missed opportunity for substance use treatment centers to comply with clinical care guidelines [11, 12] and to intervene to reduce tobacco use and related health disparities among their clients.

There are several previously identified barriers to providing tobacco cessation treatment at substance use treatment centers, including limited training, limited resources, time restraints, and cultural norms [9, 17–24]. Additionally, available treatment opportunities that take little time or training, such as referral to a state tobacco cessation quitline, are often unknown by staff at substance use treatment centers [23]. Our use of “staff” here refers to both clinical employees, those providing direct services to clients, and nonclinical employees. Moreover, despite evidence to the contrary, staff may believe that treating tobacco use and substance use disorders simultaneously will jeopardize substance use treatment and recovery [25]. Together, these barriers and others may contribute to the known translational lag whereby any type of evidence-based practice takes a long time (e.g., up to 17 years) to be implemented into practice to reach the intended population and ensure the improvement of clients’ health [26, 27]. While this translational lag is detrimental for all clients and communities, the negative consequences of these delays are even worse for populations who experience health disparities, such as individuals living with substance use disorders.

Together, the previously described evidence-based tobacco cessation practices and policies, such as tobacco use assessment, brief cessation advice, individual or group counseling, pharmacotherapy, tobacco-free policies, form the core components of a comprehensive tobacco-free workplace program [28, 29]. Academic-community partnerships can assist substance use treatment centers in implementing comprehensive tobacco-free programs and reducing the translational gap that affects health disparities among their clients [29–32]. This study describes the implementation of a tobacco-free workplace program at a substance use treatment center in Houston, Texas, which included a comprehensive tobacco-free workplace policy implementation, education and specialized training support, and the provision of resources to support tobacco cessation care. The goal of the study was to identify barriers and facilitators to successful integration of tobacco-free workplace policy and cessation practices into a substance use treatment center. The current study was based on an ethnographic approach and uses a case study design, which is considered an efficient way to present qualitative ethnographic findings [33, 34]. Case study design has been found to be particularly useful in implementation research, as it allows for an in-depth analysis of complex interventions in combination with a participatory approach in a real-life context [35–38]. Prior research has shown the importance of studying interventions in close connection with the context of dynamic environments that can have an extensive influence on the implementation process [37]. In the case of complex interventions, such as comprehensive tobacco-free programs, this is particularly relevant, given their dependence on contextual elements for their effectiveness [39]. Intervention and context cannot be easily separated in this situation, and there is an urgent need to better understand the relationship between these two core elements of implementation to ensure that research evidence can meaningfully impact policy and healthcare organizational culture [38]. By applying a case study design, this work contributes to the existing research on implementing tobacco-free workplace programs at substance use treatment centers [32] by providing an in-depth qualitative description of program implementation in the setting of a nonprofit outpatient substance use treatment center serving diverse clients, most of whom belong to socioeconomically disadvantaged and medically underserved groups. Additionally, this study represents both staff, clinical and nonclinical alike, including leadership, and clients’ perspectives on this program, the latter of which were not included in prior work [32]. The findings presented in this study can be used by other substance use treatment centers that serve similar populations and seek to implement a comprehensive tobacco-free program in the most sustainable way.

### Case description

This initiative was undertaken as a part of the Taking Texas Tobacco Free (TTTF) program, which is a multicomponent, evidence-based comprehensive tobacco-free workplace program that was designed to address tobacco dependence within healthcare treatment settings, including substance use treatment settings [30, 32]. TTTF includes (1) tobacco-free policy development and implementation and/or refreshment for comprehensiveness or quality assurance; (2) education and specialized training for staff on tobacco use and cessation, screening practices, and treatment provision; and (3) resource provision, including free nicotine replacement therapy, signage, and passive dissemination materials. Throughout the implementation process, TTTF team members, comprising an academic-community collaboration, provide ongoing technical assistance and support (for more information on the TTTF program, see previously published studies [28, 30, 31, 32, 40–44]).

To ensure the privacy of the research participants, we refer to the field research location as the “Center” herein. The Center is located in a Houston, Texas, zip code that is among the Centers for Medicare and Medicaid Services-designated low-income and health professional shortage areas. It is a small Center that employs 7 staff (including clinical and nonclinical staff) and serves approximately 1,000 unique clients each year. One of these staff members was designated the TTTF program champion to serve as the main point of contact for all aspects of the tobacco-free workplace program implementation process. This staff member was not financially compensated for accepting this role, but they received additional week-long full-time training to become a Tobacco Treatment Specialist. The financial compensation for this role was not a part of the current program, and the expectation was that the Center’s leadership incorporates this role in the regular scope of work for their staff to ensure the sustainability of the program.

The Center serves a diverse group of clients, with 90% of their clients having histories of incarceration or another form of engagement in the criminal justice system, many of whom come from low socioeconomic backgrounds and/or have been diagnosed with comorbid behavioral health (i.e., mental health or substance use disorders) and physical health conditions. The Center estimated that approximately 80% of their clients smoked conventional cigarettes and 30% used other tobacco products, including e-cigarettes (there is an overlap, as some clients might be dual or multiple product users). Most clients participate in the Center’s substance use treatment program for 90 days. The Center introduced a tobacco-free policy in 2000, which prohibited the use of tobacco products of any type both indoors and outdoors; however, they had not provided any tobacco cessation services to their clients beyond the requirement not to use tobacco on their property prior to their enrollment in the TTTF program. The tobacco-free workplace program implementation components and the Center’s timeline are presented in Table 1.

**Table 1:** The Taking Texas Tobacco Free (TTTF) Program Components and Major Implementation Milestones at the Center (a Substance Use Treatment Center in Houston, Texas)

Program Component	Date	Comments
Signing the Memorandum of Understanding	August 2021	Required to demonstrate CEO commitment to participate in the program (includes expectations regarding staff and leadership participating in the program, time commitment, etc.).
Program champion attended a 5-day course to become a Tobacco Treatment Specialist	September 2021	Program champion is one of the Center’s staff members designated by the leadership to serve as the main point of contact for all aspects of the tobacco-free workplace program implementation process. This person was employed at the Center as a Community Outreach Specialist and was not financially compensated for accepting this role to oversee the program implementation at the center.
A 1.5-hour long training on the harms of tobacco use and treating tobacco dependence provided to all staff	October 2021	Provided as a part of the Center’s annual retreat; training was provided in person by one of the TTTF staff members and the Center’s program champion.
The Center developed several documents in close cooperation with TTTF staff	September– October 2021	Documents included: updated tobacco-free workplace policy, tobacco use assessment forms, and nicotine replacement therapy storage policy.
Center received a shipment of nicotine replacement therapy products, including gum, patches, and lozenges; intended to be a starter kit that the Center would supplement over time from their own budget or grants/donations	October 2021	These products were provided to the Center free of charge, with costs covered by the TTTF program (total cost $3,369.72 for 156 boxes of nicotine replacement therapy products). The amount of products needed was estimated by TTTF staff in cooperation with the Center’s leadership based on the number of clients they served and their tobacco use rates. The second shipment of nicotine replacement therapy products was requested by the Center and provided free of charge, with costs covered by the TTTF program in July 2022 (total cost $2,968.88 for 132 boxes).
Center received new signage to place indoors and outdoors to inform staff, clients, and visitors about their tobacco-free policy	October 2021	Signage included 2 outdoor metal signs, 1 window cling, and 4 indoor paper signs, and was provided to the center free of charge.
Center received dissemination materials, including posters to display on the walls and information cards to give out to clients	October 2021	Posters and information cards were also provided free of charge and covered various areas of smoking cessation, ranging from general information on the harms of smoking to more specialized information on tobacco and substance use, and tobacco use and mental health, etc. They were selected by the Center’s leadership from a variety of options provided by the TTTF program to best reflect their clients’ needs and demographic characteristics.
Center-wide quit “refresh” date	January 3, 2022	The quit date is defined as the date the policy becomes effective; this policy “refresh” date was selected by the Center.

## Methods

This project was approved by the Internal Review Board of the University of Houston and the Quality Improvement Assessment Board at the University of Texas MD Anderson Cancer Center. Oral informed consent was received from all participants prior to participation in qualitative study procedures. The aims of the project and interviews were discussed with participants who were given an opportunity to ask any questions about the interview process and the nature of the study. Additionally, all participants gave oral permission to audio-record the interview; they were given the option to remain anonymous and not use their names or other identifying information in any written summary of the collected data. Participants were informed that their participation was voluntary, that they could decline to answer any questions and stop participating in the interview at any time.

### Data collection instruments

Data for this qualitative case study were collected via group and individual interviews with staff and clients at the Center, as well as site visits and participant observations. Data include interview transcripts and fieldnotes. We conducted one pre- and one post-implementation focus group with clients (*n* = 16), two pre-implementation semi-structured interviews with staff (*n* = 2), two individual interviews with staff during the implementation process (*n* = 2), and one post-implementation group interview with staff (*n* = 2). Interview guides were used for interviews and focus groups, which lasted 60–90 min (see Additional file 1: Interview Guides). Pre-implementation interview questions for staff focused on any Center-specific needs for the program rollout, populations they served, their personal experience with tobacco use, their knowledge of and attitudes toward tobacco use and cessation among their clients, and implementation barriers and facilitators they anticipated. Staff post-implementation interview questions addressed experiences with implementing the program, interventions that were successful and less successful, changes in their practices addressing tobacco dependence, and any challenges they experienced. Focus groups with clients addressed their experiences with tobacco use and cessation, their knowledge of the tobacco-free program at the Center, their attitudes toward and interest in this program, and their experiences with and results of receiving any tobacco cessation support at the Center. In addition, we undertook several site visits (*n* = 8), when A.R. (the 1st author) and I.M.L. (the 2nd author), both cultural anthropologists who worked as qualitative research specialists on the project, conducted observations and made fieldnotes using a free-form approach. The site visits (1 to 2 h long) incorporated both direct and indirect observations. The collected observational data were not subjected to standalone analysis but served to inform the interview questions, gain a more nuanced understanding of how the Center was implementing various parts of the program, and provide further details about the study’s context and setting.

Two authors (A.R. and I.M.L.) moderated the focus groups and completed the interviews. Audio-recordings of focus groups and interviews were transcribed verbatim by a professional transcription service and analyzed using thematic analysis to initially inductively code and identify themes within the dataset. Data analysis was conducted iteratively using constant comparison, and themes were drawn directly from the data. The process of constant comparison provided analytic rigor and ensured accurate accounting of all the data, identifying appropriate selection of categories and themes [45]. At the next stage of the analysis, the concepts of the extended Normalization Process Theory (discussed in detail below) were applied to these themes to more effectively analyze and evaluate the implementation process.

### Approach: extended normalization process theory

When exploring the implementation process, the application of a theoretical framework enhances understanding of the process and highlights barriers to and facilitators of the implementation. Implementation scientists have developed several major frameworks and theories to describe and evaluate the implementation process [26, 46–49]. For this analysis, we followed the extended Normalization Process Theory (eNPT) [46, 47], which is a sociological theory that informs the implementation of innovations in healthcare practice, focusing on bridging the translational gap between evidence-based practices and their implementation [50]. This theory approaches the implementation process as a series of interactions between people’s actions (their ‘agency’) and the context within which the intervention is implemented [46, 50]. 

**Fig. 1:**
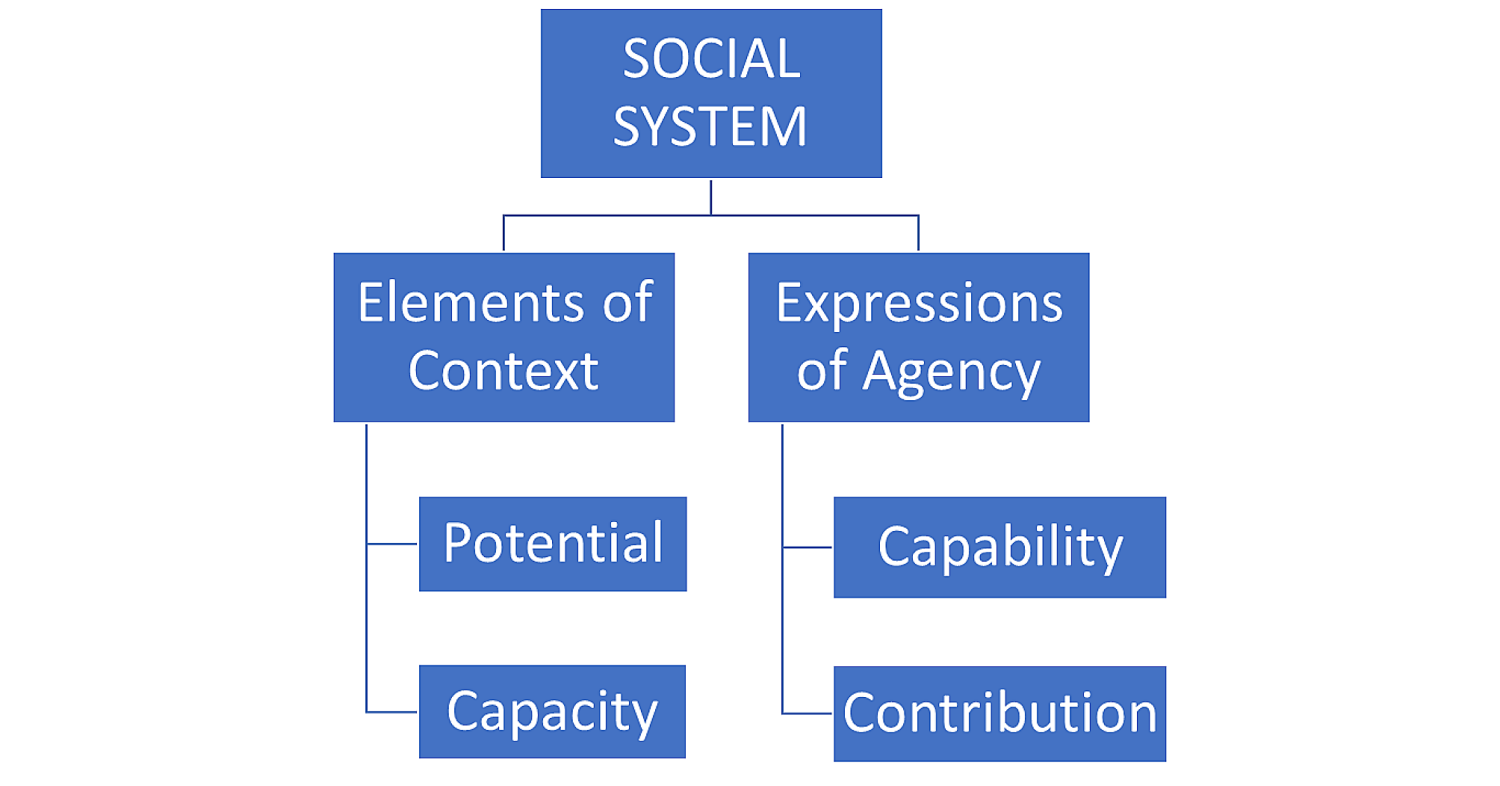
Concepts and Constructs of the Extended Normalization Process Theory (eNPT, adapted from May 2013 [46])

The eNPT identifies and explains key elements that contribute to or impede normalization of complex interventions within a social system, including four core constructs, two of which are focused on context (potential and capacity) and two of which address agency (capability and contribution, see Fig. 1 for details) [46]. The identification of these major constructs helps researchers guide and understand the implementation process and provide a systematic description [46]. The eNPT has been effectively utilized at all stages of research projects, both during the planning stages and the post-implementation analysis, as in this case, where this theory helps to frame emergent themes and consider their implications for further research and implementation practice [51]. The application of the eNPT as a theoretical framework enhances stakeholders’ ability to improve design for more successful implementation in the future and to enhance the application and normalization of interventions within organizations by community adopters and researchers [50]. 

The two constructs of the eNPT that characterize the context of implementation are *potential* and *capacity*. Participants’ potential is expressed through individual intentions and collective commitment to participate in the intervention [52]. Capacity, which is another crucial element of context, is defined as the availability of material and cognitive resources, as well as existing social norms and social roles. Attention to these contextual elements ensures a better understanding of the implementation process and its outcomes, as they shape agents’ ability to effectively cooperate with each other to bring about change [46]. *Capability* and *contribution* are two constructs that characterize the agency of the participants involved in the implementation process. Capability refers to how workable the complex intervention is, as well as to the possibility of integrating it in everyday practice. Contribution refers to the actions of the agents who are involved in the implementation process [46]. This construct focuses on how the agents, including individuals and groups, enact potential and capacity by undertaking actions to make things happen and ensure that new processes and practices become “the way we do things here.” [46] Each of these constructs is further divided into categories used to understand and evaluate the implementation process (see Table 2).

## Results

After conducting the initial coding, the emerged themes were systematized and organized in relation to the major concepts and constructs of the eNPT theory. Table 2 shows the identified themes from the case study data and how they are related to the eNPT constructs and dimensions. In the section below, we present our results in relation to these theoretical constructs.

**Table 2:** Codes and Categories Related to the Evaluation of the Taking Texas Tobacco Free (TTTF) Program Implementation at the Center (a Substance Use Treatment Center in Houston, Texas)

Extended Normalization Process Theory (eNPT) Concepts and Categories			Case Study Themes
Context	Potential	Individual Intentions	- Mostly nonsmoking staff/negative experiences with smoking related to health concerns - Overall support of the TTTF program
		Collective Commitment	- Expressed readiness to participate in the program/provide services to the clients - Commitment to support clients and motivate them to quit tobacco use - Leadership support
	Capacity	Social Roles	- Professional roles and expectation of client-clinician interaction
		Social Norms	- Clients’ experiences with tobacco and smoking cessation - Clinicians’ attitudes and expectations
		Material Resources	- Nicotine replacement therapy products provisions
		Cognitive Resources	- Trainings
Agency	Capability	Workability	- Familiarity (existing tobacco-free policy) - TTTF program support - Program champion role
		Integration	- Integration into everyday practices
	Contribution	Coherence	- Seeing the value
		Cognitive Participation	- Community of practice - Suggestions for improvements
		Collective Action	- Change in practices - Enforcement of policies - Regular trainings - Staff quitting smoking - Lack of consistency/clear guidelines
		Reflexive Monitoring	- Need to do more - Suggestions for improvement - Perceived value of the program

### Context: potential

The Center’s CEO initiated the Center’s participation in the tobacco-free workplace program and continuously expressed their personal support and commitment to implement and maintain the program. The Center’s staff were also enthusiastic about the tobacco-free program and expressed commitment to its implementation. Most participants welcomed this forthcoming change and expansion of tobacco cessation services and agreed that it was necessary and beneficial for the Center’s working environment and for their clients’ needs and well-being. This collective commitment was supported and reinforced during the preparation phase of the implementation process, when all staff received training provided by the TTTF program:

*I think one of the biggest things in preparation for implementing the program was when we had our staff retreat. Bryce [B.K.] actually flew in, and he participated in the retreat and helped train our staff prior to us actually implementing the program. So, he did a workshop with us, and that gave us an opportunity to ask him questions […] So, everybody was ready. Everybody was pumped and ready because we knew what to do. We knew what the problem was about*. (Staff post-implementation interview)

The number of staff who used tobacco products themselves was very low at the Center. The only staff member who said that she smoked cigarettes participated in the program herself and, at the time of the interview, reported a successful quit attempt and being tobacco-free. In the pre- and post-implementation interviews, staff members shared their negative attitudes towards smoking related to health concerns and their readiness to facilitate tobacco cessation efforts at their center:

*I grew up as an athlete, and so smoking is something that was not encouraged in my field. I didn’t partake when I was around people that did such as my mom who later on in life actually stopped smoking.* (Staff pre-implementation interview)

### Context: capacity

One of the major barriers to the implementation of the tobacco-free workplace program at the Center was associated with the widespread practice of tobacco use among the Center’s clients and within their immediate environment. There are two closely interrelated aspects of this problem: clients’ lived experiences with tobacco being an innate part of their everyday life and staff attitudes and expectations of their clients’ interest in and ability to quit tobacco, both of which are discussed below.

Most of the Center’s clients grew up in an environment where smoking had been normalized for years. They shared their experiences of having parents, older siblings, grandparents, neighbors, and friends who had smoked on a regular basis for most of their lives:

*Kind of like I think started smoking from– because I would light cigarettes for my daddy or whatever, so he was smoking.* (Client focus group, pre-implementation)

*Me growing up around my grandfather and stuff, he smokes. […] Mine started just with social. Got out of high school, my own place. All my friends, they bring over rum […] and cigarettes and everything.* (Client focus group, pre-implementation)

Some of the clients reported a lack of interest in quitting or their perceived inability to quit:

*So officially, once I turned 18 or the age to buy a pack of cigarettes, I bought a pack of cigarettes and since then it’s been– I never had the urge to quit, never tried to quit. Just always adapted to it.* (Client focus group, pre-implementation)

*They [clients] point-blank told me that they feel that if they stop smoking, that they’ll latch back onto something else that is not as legal.* (Staff post-implementation interview)

However, these experiences do not mean that none of the clients problematized tobacco use practices and were interested in quitting tobacco. Some clients reported varied attitudes toward quitting. For example, one of the clients who participated in a focus group shared her motivation to quit smoking:

*I didn’t want him [her son] to - when he’s picking up pieces of paper towel and putting it to his mouth like a cigarette, it bothers me. I don’t want him to […] also, my baby’s father wasn’t a smoker. It’s a shame thing. I was ashamed.* (Client focus group, post-implementation)

In the interviews with the Center’s staff members, they generally revealed that tobacco cessation treatment was a low-priority problem, related partly to a perceived lack of interest in quitting among their clients. Staff shared expectations that clients must be proactive in expressing their interest in quitting and seeking support. Staff at the Center repeatedly expressed the idea that if their clients were interested in quitting tobacco use, they had to ask for help to proactively demonstrate that they were interested in and committed to quitting. As the Center’s program champion said during a conversation with one of the researchers:

*I see some of the guys who signed up for the program, but they go out and smoke with other guys outside. I walk by, I see him, but I am not going to say anything to him. It must be his decision, he is an adult, and he must take responsibility. I cannot do it for them.* (fieldnotes, conversation with program champion, May 2022)

In a similar way, one of the clinicians at the Center shared during the interview:

*I guess if they ask me, if they would like the patches, if they want to participate, I guess that’s when I’ll bring it up* […] *I think it’s ultimately really up to the client if they really want to make that change. That’s what I love. Some of the clients really want to commit strongly about making the change to stop smoking.* (Staff post-implementation interview)

Tobacco education trainings were offered to all Center staff at the beginning of implementation, which were designed to mitigate these barriers (e.g., by providing information about how to proactively address tobacco use with clients) and enhance the implementation capacity by ensuring that they had the knowledge and skills required to implement the program. One of the staff evaluated this training as being very important to help them to be able to deliver tobacco cessation services:

*Teaching us about pharmacology, motivational interviewing […]. That thing that was really helpful for us to learn and to be able to explain it to the clients if questions were to come up.* (Staff post-implementation interview)

The capacity to implement the program also depends on the availability of material resources. One of most important and expensive resources, nicotine replacement therapy products, were provided to the Center free of charge as a part of the active implementation process. The availability of the nicotine replacement products was widely discussed by the Center’s staff and evaluated as one of the central elements of the program implementation at the Center:

*We actually not only have “No Smoking” sign posted up, but we’re able to say, “Here, we have products, nicotine replacement products, that we could give you to help you stop smoking.”* (Staff post-implementation interview).

### Agency: capability

The Center’s capability to implement the program was evaluated by assessing the implementation’s workability and integration with the everyday workflow and preexisting work processes, following the eNPT framework concepts.

The Center already had a standard tobacco-free policy in place prior to the involvement in the project, and while the TTTF presented them with a much more comprehensive program, the initial buy-in was facilitated by the level of familiarity with the intervention by both staff and clients:

*We weren’t really implementing anything. It [tobacco-free policy] was there. It was understood, but this gave us a fuller picture of a way to implement, how to introduce it, a guideline to follow*. (Staff post-implementation interview)

One element of the program that contributed to the increased workability was the introduction of the program champion role into the program:

*I think the best thing is to have a point person. Because we have a point person, that point person stays on top of all the policy procedures, regulation, inventory, whatever we have going on.* (Staff pre-implementation interview)

The Center’s CEO and staff also emphasized that the support they received from the TTTF program increased the workability of the intervention. In addition to regular practical and informational support, they were able to contact program staff with any ongoing questions and requests for assistance. They shared that the focus groups that were conducted with clients also contributed to the program implementation success by increasing clients’ interest in the program:

*You guys come in here and working with those guys, because you legitimize the process as a third-party source, and the guys come in to see and you do the surveys* [focus group] *with them. I think that’s very helpful.* (Staff post-implementation interview)

New aspects of the program were reported to be well integrated into the everyday workflow, and while their implementation needed certain changes in practices and attitudes among staff and clients, these changes were not particularly disruptive or time consuming, according to staff who participated in post-implementation interviews:

*It’s good to have it embedded into the program that you already have, immerse into what you have going and make it a part of the process, not as something separate, but just this is our program. This is included in the program. I think it’s welcomed a little bit more.* (Staff post-implementation interview)

### Agency: contribution

Most staff members shared a clear understanding of the purpose of the intervention and its potential benefits. They evaluated the program as important, saw the value of this program for their clients, and shared positive experiences of being involved in its implementation:

*I love the program because it gives the clients an opportunity to work on solving that problem of addiction in a positive manner.* (Staff post-implementation interview)

*I felt like it was a great idea to come into play here at the facility.* (Staff post-implementation interview)

All staff were well aware of the program being implemented and what new practices and routines were introduced at the Center. They reported very little disagreement about a shared understanding of the need to implement this program:

*Everybody was clear on what the mission was, how we would present it, and the way it would be implemented*. (Staff post-implementation interview)

One example of effective engagement with the program was one of the staff members quitting smoking herself:

*When I came in, he was doing a class and I sat in on it and I’m like, “Hey, I want to do this.” […] we talked about it and I signed up to do it. It’s worked very good for me.* (Staff post-implementation interview)

In the interviews, staff at the Center discussed how their engagement in the program and enacting it in their everyday practices contributed to their deeper sense of belonging:

*I have the feeling like you can’t disrespect the facility [*by smoking*]. This is our facility and we need to respect her.* (Staff post-implementation interview)

Staff members shared a commitment to serving their clients and supporting each other, which was further reinforced by their increased capacity to provide tobacco cessation support to their clients:

*That sign right there says we are community, and that’s what we promote, that we are a community center, and this community center has many different programs in it that can provide assistance and this is one of the additional programs that we have that can provide assistance.* (Staff post-implementation interview)

Various program components were implemented with different degrees of commitment. The tobacco-free policy was the component that staff reported to be implemented most consistently. The tobacco-free policy has been routinely maintained and reinforced by both the Center’s staff and clients themselves:

*They are not allowed to smoke within the facility area. So, that’s worked pretty good.* (Staff post-implementation interview)

*We were always like, “You can’t smoke in here. You got to walk outside.” I think that they just pretty much are just like, “Okay, we got to do the right thing.” […] So yes, they respect it, I think*. (Staff post-implementation interview)

However, as A.R. and I.M.L. observed when they visited the Center, clients were often smoking outside. While they were not violating the policy as they were technically outside the property and were smoking while on a public road, they remained physically close to the building, and whoever was leaving or entering the property had to go past a group of clients smoking to enter through the only door to the Center. The Center’s leadership has not found a solution to this problem, as they said they did not have control over the territory and could not prohibit tobacco use beyond their property. This location-specific issue led to a situation in which the tobacco-free policy was technically enforced; however, clients were still able to smoke in the vicinity of the Center, visitors were exposed to secondhand smoke, and this practice was not challenged by leadership or staff, either pre-implementation or after.

Tobacco screenings were reported to be implemented on a regular basis, although there were some discrepancies in the participants’ accounts of screening practices and their regularity. All clients were reportedly screened for all forms of tobacco use during intake, but the follow-up screenings of those clients who reported using tobacco were less consistent. There seemed to be a lack of clear understanding and agreement among staff who was responsible for these screenings, which resulted in a lack of consistency and depended on a specific staff member’s practice rather than established and clearly understood guidelines:

*Each time we do an intake on a form, there is an assessment that asks the client if they do smoke, and if they do smoke, do they smoke cigarettes, or do they smoke e-cigarettes? We do offer the NRT [nicotine replacement therapy, and if they want to participate, they would need to say yes or no. Let the counsellor know. […] The individual counsellors, after 30 days in their sessions, ask them again.* (Staff post-implementation interview)

However, in individual interviews with staff, at least one of them said that they did not conduct any follow-up screenings unless their clients brought this up and asked about the tobacco cessation program themselves.

As one of the central elements of the program implementation, the program champion provided regular information sessions to inform their clients on the Center’s participation in the program and available support and resources for clients who were interested in quitting tobacco. All clients were expected to attend at least one of these sessions, as these presentations were performed during their mandatory group counseling sessions. Clients were made aware of the resources and support available to them at the Center if they decided to make a quit attempt, as well as given a brief educational presentation on the harms of smoking and the benefits of quitting. These presentations were seen as an effective tool to get clients interested in the program, provide them an opportunity to ask for more information, and engage in conversations about quitting:

*People have changed their minds, actually. They initially said no, but then once they heard* [the program champion] *and people talk about it, they come back and say, well, yes, they would like to. There’s been a couple of guys that have done it, that I know personally, that have done that.* (Staff post-implementation interview)

The actual engagement of clients and motivating them to make a quit attempt was the most challenging part of the implementation process for the Center. The overall number of participants who made a quit attempt was 17 clients and two staff members by the end of the implementation period. While the reach of the program is larger than immediate client participation in cessation treatment, there were also some clients’ accounts of inconsistency in support they received during their time at the Center regarding their tobacco use:

*Nobody has ever asked me anything [about tobacco use], except you.* (Client focus group, post-implementation)

While staff supported the implementation of the program from the beginning, there were some concerns about how well this program might be accepted by their clients. In the post-implementation interview, a counselor shared an observation that their clients were more interested in quitting than they anticipated:

*I guess I’m just surprised that I feel like I’m getting some yes’s now instead of a whole bunch of no’s. So, I think that’s actually a good thing because I feel like now that the program has been implemented here, that we’re getting quite a few yes’s. So, that’s definitely something to feel good about, that makes me feel good.* (Staff post-implementation interview)

While we observed a variation in the degree to which tobacco cessation intervention services were provided in practice, there was a shared understanding that some of the services needed to be improved:

*To be honest, it’s a question* [tobacco use and interest in quitting] *I feel like I need to ask them more. I haven’t been asking them about it, but I feel like I do need to ask them*. *[…]So, that’s something I could work on.* (Staff post-implementation interview)

In the quote above, the counsellor acknowledges that they should ask their clients about their smoking habits and interest in quitting more proactively, which is a positive example of reflexive monitoring of their own actions and practices and could ultimately lead to better outcomes of the intervention.

Staff also demonstrated their involvement by critically evaluating the program delivery and expressing suggestions for improvement:

[We say]*“We’re going to have smoking cessation group today and this is going to be the only one for the month.” Well, why can’t we bring it up every meeting? Look, we have three meetings a week, let’s bring it up every time. […] I think there should be a smoking class […] for the whole group at least once a month.* (Staff post-implementation interview)

Staff reflected on how this program changed their Center, and they reported a positive change, creating an opportunity to provide more meaningful and involved support and services to their clients:

*It’s positively changed or impacted our facility because it gives us some legitimacy behind not only just having a no smoking sign just posted like every public place you see, but actually giving some type of support, nicotine replacement therapy. […]* (Staff post-implementation interview).

## Discussion

This case study discusses the implementation of a tobacco-free workplace program at a substance use treatment center serving a diverse group of clients, including many from low socioeconomic backgrounds. This analysis and consideration of the interplay between context and emergent agency, facilitated by the application of the eNPT framework, contribute to the existing knowledge on implementing similar programs in substance use treatment settings that serve marginalized and medically underserved populations facing socioeconomic and health challenges. The findings from this study offer insights that can guide other substance use treatment centers with similar populations in implementing sustainable tobacco-free programs effectively.

A key barrier associated with the context of the implementation, as defined by the eNPT framework, was the widespread tobacco use among clients and within their immediate environment. Prior research has indicated that individuals with substance use disorders are often interested in quitting smoking [16], but they tend to have lower success rates [53, 54]. These contextual barriers to achieving success in tobacco cessation efforts among this population require an exceptionally high level of commitment from the staff working at substance use treatment centers to provide continuous, robust support to their clients [55, 56]. As our findings suggest, it is essential to acknowledge and consider the difficulties faced by these individuals when they are trying to quit smoking. While these challenges should not deter clinicians from motivating their clients to quit smoking, it is crucial that they are prepared to approach the situation with sensitivity and awareness of the contextual factors and lived experiences of the clients, which is also emphasized in the principles of trauma-informed care.

Other contextual categories, defined by the eNPT, which we addressed in our study to evaluate the Center’s potential to implement the program, include individual intentions and collective commitment shared by staff and leadership. Most of the staff expressed a strong commitment to participate in the program and provide cessation services to the clients. However, we also encountered attitudes indicating that staff, including clinicians, were doubtful about their clients’ interest in quitting and ability to do so. Given their expertise and supportive roles as addiction treatment specialists, clinicians’ attitudes can greatly affect those of their clients; moreover, clinicians’ beliefs and attitudes are often cited as one of the major barriers to effectively implementing tobacco-free programs within substance use treatment settings [4, 22, 25, 57]. Training given as a part of the program implementation provided staff with information on evidence-based tobacco cessation practices and addressed some of these attitudes to better prepare staff to provide cessation care to their clients. Such training programs are particularly important for successful implementation and can be further enhanced by placing a stronger emphasis on motivational interviewing techniques, providing practitioners with a better understanding of the nature of ambivalence toward behavior change and the diverse factors influencing clients’ readiness to quit tobacco use.

The capacity to successfully implement and maintain the tobacco-free workplace program is also dependent on access to material resources and, specifically, nicotine replacement therapy products. While two shipments of nicotine replacement products were provided free of charge by the TTTF program, ensuring a continuous supply of these products is anticipated to be challenging for the Center. While individual clients can access free nicotine replacement products through services such as the Texas Tobacco Quitline [58], the availability of these products on-site and the ability to distribute them immediately and at no cost has been emphasized by the Center’s staff as a crucial component of the program. To address this challenge, the TTTF staff provided informational resources to the Center’s leadership and program champion, highlighting the support available in the community to secure additional funding for the ongoing purchase of nicotine replacement products. However, it remains uncertain at this stage whether the Center will be able to secure the necessary funding to sustain the provision of free nicotine replacement products to their clients and how the availability of these products will impact the long-term sustainability of the tobacco-free program. This is a limitation of this study, as it was conducted during the active phase of implementation and shortly after its completion, lacking data on the program’s long-term maintenance and outcomes. Therefore, further investigation specifically focusing on the long-term sustainability of tobacco-free programs at substance-use treatment centers would be valuable to address this gap and provide insights into ensuring ongoing access to nicotine replacement therapy products for patients. We suggest, however, that it is important to maintain communication with centers after the program implementation is completed, highlighting specific local funding opportunities, as well as sharing examples of successful programs maintained by other centers as a mechanism to support collaboration and pursue additional resources.

Analysis of the themes reflecting the expressions of agency, another major eNPT concept, showed a gradual positive change in tobacco treatment practices at the Center following the implementation of the program, including the enforcement of policies and staff quitting smoking. However, the findings also show that these changes did not immediately affect the provision of smoking cessation care to clients at the Center. Tobacco cessation treatment remained a problem of a lower priority, even for staff who had negative experiences with smoking associated with health concerns, did not use tobacco themselves and were overall very supportive of the program and excited about helping their clients to quit. Rather, this seemed closely related to a persistent perception that their clients were not genuinely interested in or capable of quitting, which was also revealed in the expectations shared by staff that clients had to be proactive in expressing their interest in quitting and seeking support.

We suggest that the expectation of clients proactively seeking support shared by the Center’s staff is associated, at least partially, with their understanding of the existing standards of client-clinician communication, which emphasizes the importance of “sharing power” equally with clients and involving them in the decision-making process [59]. The concept of patient-centered care, designed to improve healthcare provision and outcomes, is often regarded as a matter of ethical and moral healthcare practice, and it assumes patients’ involvement in their care [60, 61]. It is important to consider, however, that these expectations might not work as planned with vulnerable populations, including clients who experience socioeconomic disadvantage, limited access to healthcare services, lower literacy levels and/or limited English proficiency [62]. These individuals’ ability to take a proactive stance and advocate for their health and well-being may be further hindered by systemic inequalities and structural racism disproportionately experienced by minoritized and underserved groups, and these factors have to be considered to improve the delivery of patient-centered care to these clients and ensure that the care they receive is tailored to their specific needs. Taking Texas Tobacco Free program has developed multiple training videos on smoking cessation support to special population groups [63], which can be used to provide continuing education on working with diverse groups to ensure that healthcare professionals are equipped with knowledge and skills needed to provide such care.

It is important to acknowledge that concerns about promoting smoking cessation are not entirely unfounded, as clinicians’ advice can have various consequences beyond the client simply following or not following it [64], and prior research has shown that avoidance of hearing specific recommendations to change behavior, including smoking, is reported as one of the reasons why people avoid seeking medical care [65]. However, these findings should not discourage health care providers from asking their clients about tobacco use, as this practice is associated with increased quit attempts and is recommended by The US Public Health Service Clinical Practice Guideline [66]. The potential risk of inadvertently stigmatizing clients who may already feel shame and guilt regarding their tobacco use and inability to quit might be avoided if clinicians use non-stigmatizing approaches identified in prior research [67–71]. It is particularly important to address these concerns in tobacco cessation trainings and educate staff on non-stigmatizing approaches. It is important to incorporate motivational interviewing in these trainings as this approach highlights the importance of displaying unconditional positive regard toward clients, which may increase client resilience in the face of behavioral change advice offered and minimize perceived stigma. It is crucial to find a balance between being sensitive to clients’ choices and priorities and providing the healthcare necessary to alleviate the consequences of systemic health inequities among minoritized and medically underserved groups.

One of the limitation of the study is the limited data on clients’ quit attempts and their outcomes. While the Center attempted to collect these data, they had difficulties following up with their clients after they left the program (most of the clients attended a 90-day program), which created difficulties in evaluating outcomes of those clients who initiated a quit attempt while being treated at the Center. While a more detailed analysis of client outcomes would enhance the evaluation of the intervention, the focus of this study has been on the implementation outcomes, including changes in provider behavior regarding assessing and treating tobacco dependence rather than assessing its direct impact on clients’ tobacco use and cessation [72]. Future research is needed to delve into evaluating the effects of the intervention on clients’ outcomes, which would provide valuable insights for further refining and optimizing the program.

While the Center’s staff exhibited strong potential and capacity to implement the program, our findings indicate that the actual change in practice has been less successful than anticipated based on the overall support of the program, high potential, and capability. Tobacco cessation treatment had not yet become a routine practice for all staff members by the end of the implementation process. However, despite encountering significant barriers, there is evidence that the program has led to a change in attitudes, including a better understanding of the need and improved ability to provide evidence-based tobacco cessation treatment to their patients. The staff at the Center have started to integrate tobacco treatment into their routine practices, informing clients about the available support, including nicotine replacement therapy products, providing personalized assistance, and assessing patients who may not be ready to quit. Although there are areas for improvement, the program has effectively initiated change in practices, normalizing tobacco cessation treatment and incorporating it as a routine practice at the Center.

## Conclusion

The results of this study suggest that substance use treatment centers can maintain tobacco-free workplace policies and integrate evidence-based tobacco cessation practices in their daily operations, but they face extreme challenges due to the complex behavioral health needs and socioeconomic needs of their clients. Understanding the complex interplay between social norms, social roles, and limited resources within such settings is paramount for the success of tobacco cessation efforts. These organizations need extensive support, including a longer implementation period, as well as additional material resources, informational and educational support, and assistance in preparing and maintaining local policies. Regular training of staff, including implementing a train-the-trainer program, would allow to promote and sustain local expertise on evidence-based tobacco cessation interventions for minoritized and medically underserved populations. With proper support, substance use treatment settings have the potential to play a crucial role in addressing tobacco use and provide much needed cessation services to their clients who are disproportionately affected by tobacco-related health conditions and systemic health inequities.

### Electronic supplementary material

Below is the link to the electronic supplementary material.

Supplementary Material 1

## Data Availability

The datasets used and/or analysed during the current study are available from the corresponding author on reasonable request.
