# Supplementary material for: Implementing a tobacco-free workplace program at a substance use treatment center: a case study
Source: BMC Health Serv Res. 2024 Feb 14;24:201. doi: 10.1186/s12913-024-10629-5 (PMC10865640; doi:10.1186/s12913-024-10629-5)
Supplement: Supplementary file 1 — Supplementary Material 1 [file 12913_2024_10629_MOESM1_ESM.docx]

**Taking Texas Tobacco Free among Substance Users within Community-based Healthcare Settings in Rural and Medically Underserved Areas Across Texas**

Pre-implementation Interview Guide for Clients

1. What has been your experience with smoking?
2. How long you have been smoking?*
3. How many cigarettes are/were you smoking per day?
4. What kinds of tobacco products do you use? (cigarettes, e-cigarettes, dip)
5. If you don’t smoke, how do you feel about other people smoking?

**Here and below: these additional questions are probing questions to be asked to help the interviewee to elaborate, if additional information is not provided spontaneously.*

1. Why do you smoke?
   1. What are some benefits you get from smoking?
   2. What are the negatives about smoking?
2. How does smoking in your environment affect you?
   1. How do people you hang out with, like friends, family, influence your smoking/not smoking?
   2. What about smoking here in (name of center) where you receive services?
   3. What concerns, if any, do you have about secondhand smoke?

1. How do you feel about quitting smoking?
2. How valuable do you think it is to quit smoking? (Motivations: health, family, stigma?)
3. What has been your experience on being advised to stop smoking by friends, family, or healthcare providers?
4. If you are interested in quitting smoking, why do you still smoke? What are some of the negative and positive things about smoking that make it hard for you to quit?
5. Have you ever tried to quit smoking before? Tell me about that.
6. What made you want to quit smoking now or during past quit attempts?
7. Who supported you during your quit process? (Staff, friends, family)
8. What’s been most helpful to you in quitting? (Support, NRT, counseling individual/ groups, medications)
9. How much do you pay for your cigarettes? How much do you spend on cigarettes during a week?
10. Can you describe the atmosphere towards smoking here at (name of center)? Do they discourage or encourage smoking here?
11. How does staff react when they see you smoking?
12. How often does staff join clients to smoke?
13. What types services or help do you receive from (name of center)?
14. How do you feel about the quality and types of services (name of center) offers to quit smoking?
15. What types of help have you gotten to quit smoking? (Counseling, NRT?)
16. How often does your counselor/physician talk to you about smoking and maybe quitting?
17. What kind of education have you received about the harms of smoking and quitting?
18. If you had a chance to create a quitting smoking program, what would you like to see in your quitting smoking program? What would really help you quit?
19. How do you feel about (name of center) becoming 100% tobacco-free?
20. How do you feel about this center adopting a mandatory 100% smoke-free policy?
21. Can you tell me if you’ll continue to come here if you can’t smoke here anymore?
22. How much of a change do you think going 100% tobacco-free will make to (name of center)?
23. If you are seeking assistance with substance use, what, if any, is the connection between smoking and substance use for you?
24. How do you feel about quitting smoking and drugs or alcohol at the same time?
25. Do you have anything else you’d like to talk about or tell me today?

Post-implementation Interview Guide for Clients

1. What types services or help do you receive from (name of center)?
2. How long have you received these services at this center? Have you received similar services at other centers previously?
3. What changes have you seen at (name of center) regarding smoking in the last several months? Tell me about that.
4. Or is everything pretty much the same?
5. What do you know about tobacco-free policies here the center? What do you think about them?
6. How have these changes affected you and your smoking?
7. Have you been smoking more, less, or the same?
8. How do you feel about these tobacco-free policies?
9. How would you rate enforcement of the tobacco-free policy here at (name of center)?
10. How supportive do you think staff are of these policies?
11. How supportive do you think other clients are of these policies? Do you support these policies?
12. What are the consequences for violating the tobacco-free policies, for clients and for staff?
13. Can you tell me whether you’ve tried to quit since (name of center) made these changes?
14. What motivated you to quit or cut down? (E.g., health, costs, children?)
15. Who/what has helped you the most to quit or cut down (Counseling, physician, NRT, medications)?
16. How do you feel about quitting?
17. How does your family, friends feel about you quitting?
18. Have you tried quitting before? Was it different this time?
19. Can you describe the atmosphere towards smoking here now at (name of center)? Do they discourage or encourage smoking here?
20. Do you smoke when you are here? Do other clients smoke? Where do you smoke?
21. How does staff react when they see you smoking?
22. How often does staff join clients to smoke?
23. What other kinds of changes have you seen at (name of center) since they went

tobacco-free?

1. Relationships between clients? Between clinicians and clients?
2. What new services or procedures are being offered to help clients quit smoking?
3. For those of you in recovery from substance use, what has been your experience of quitting smoking while in recovery?
4. Do you think it’s been easier or harder for you? Why?
5. What, if any, connections have you noticed between smoking and your substance use?
6. What has been the hardest part of quitting? What has been the easiest?
7. How do you feel about the quality and types of services (name of center) offers to quit smoking?
8. What types of help have you gotten to quit smoking? (Counseling, NRT?)
9. Have you ever used NRT to help you quit? If you haven’t used them, why?
10. How often does your counselor/physician talk to you about smoking and quitting?
11. What kind of education have you received on: how smoking affects your health, quitting smoking, secondhand smoke, and tobacco-free policies?
12. Who provided you with that education?
13. How helpful did you find that information?
14. If you could design your perfect quit smoking program, what would it include? What would really help you quit?
15. What else could clinicians/physicians do to help you quit smoking now?
16. How do you feel about your provider keeping track of your smoking and quit attempts?
17. What effect – if any – did it have on your smoking? (Motivating, annoying)
18. How do you think the process could be improved to help you and other clients to quit?
19. Do you have anything else that you’d like to talk about regarding quitting smoking?

Pre-implementation Interview Guide for Staff

1. What do you think about our dissemination materials? [interview participants are shown the dissemination materials available for them to order as a part of the program implementation] How useful will they be in your setting? What would you recommend changing to better suit your facility?
   1. What other special populations or age groups, etc., would you like to see included?
   2. Aside from English and Spanish, are there any other languages that would be particularly relevant in your setting?
2. What kind of services do you provide to clients?
   1. Who provides these services?
   2. How often are these services provided?
3. Tell us about tobacco use among clients. How many of your clients smoke?
   1. What is included in the tobacco screenings of clients, if any, conducted by your center – i.e. other tobacco products (smokeless tobacco, e-cigarettes?)
   2. Do you ask about other substance use – i.e. drug and alcohol use?
   3. If your center does not conduct tobacco screenings, why not?
   4. If so, how often?
   5. Who conducts these screenings?
   6. How are these screenings documented? (EHR?)
4. What is your personal experience with smoking?
   1. Any interest or experience with quitting either in the past or now?
   2. If you are interested in quitting smoking, why do you still smoke? What are some negative and positive things about smoking that make it hard for you to quit?
5. What smoke-free policies or restrictions do you currently have in place for smoking indoors (vaping) and outdoors in your center?
   1. Have you had any complaints from non-smokers about exposure to second-hand smoke?
   2. How concerned are you about exposure to second-hand smoke?
6. What kind of tobacco education trainings have you received, either through your center or another organization?
   1. Who receives these trainings on tobacco education, and how often?
   2. What is covered in these trainings? (General harms of tobacco use? How to treat tobacco use generally or in special populations?)
   3. How long do these trainings generally last?
   4. Who provides these trainings?
   5. What do you think should be included in these trainings? E.g. e-cigarettes, secondhand smoke, smoking while pregnant?
7. What kinds of tobacco cessation services do you currently provide to clients and/or staff at your center? (nicotine replacement therapy, individual/group counseling)
   1. Is your center contractually mandated to offer smoking cessation services at your facility by the county/state?
   2. Are there ways in which you could integrate smoking cessation with other services that you provide? (For example, do you think clients’ groups could discuss smoking cessation?)
   3. Would you be able to dedicate staff to providing smoking cessation care?
   4. If you had a chance to create a smoking cessation program for your clients, what would you like to see in your smoking cessation program? What would really help clients quit smoking?
8. Are there any unique characteristics about the clients you serve and/or the issues they face that you are concerned about regarding the implementation of a tobacco-free program?
   1. How do you think these issues should be addressed?
   2. What do you currently do to help your clients who want to quit smoking?
   3. Where do you think your clients are with wanting to quit smoking?
9. How do you honestly feel about your facility becoming tobacco-free? What are your concerns about the program?
10. a. [For centers that have already adopted a tobacco-free policy] What are some things that have made it hard to sustain your center’s tobacco-free workplace policy, and what is needed to overcome these barriers? (If needed, prompt: On the level of organizational resources, clinicians, clients?)

b. [For centers that have not yet adopted a tobacco-free policy] What do you foresee will be the main barriers to adopting a tobacco-free workplace policy in your center, and what is needed to overcome these barriers? (If needed, prompt: On the level of organizational resources, clinicians, clients?)

1. a. [For centers that have already adopted a tobacco-free policy] Likewise, what have been the main facilitators to delivering and sustaining your center’s tobacco-free workplace policy? How do you think these facilitators can be enhanced/supported regarding:
   1. Does your center have any policies regarding treating clients that might support or hinder tobacco control efforts in your center?
2. [For centers that have not yet adopted a tobacco-free policy] Likewise, what do you foresee will be the main facilitators to adopting a tobacco-free workplace policy in your center? And how do you think these facilitators can be enhanced or supported?
3. Can you describe the general attitude towards treating tobacco at your center?
   1. Is addressing tobacco dependence among your clients considered a treatment priority for clinicians? If not, why not?
   2. Is addressing tobacco dependence among your clients supported or considered a treatment priority by leadership? Why or why not?
   3. Would you describe your center’s efforts to address tobacco as successful or unsuccessful, and why?
4. Smoking is the number one preventable cause of death and disability in the world. As there has been a lot of research documenting the health hazards of smoking, everyone is aware of the harms of smoking. In your experience as a clinician, what do you think drives smoking among your clients and what – if anything – do you think can and needs to be done to address it?
5. Is there anything else that you would like to share about comprehensively addressing tobacco use in your program that we have not covered?

Post-implementation Interview Guide for Staff

1. Tell me about your experience with the live-delivered TTTF trainings?
   1. Is there anything that the training didn’t cover that you would like us to include in future trainings? (E.g., e-cigarettes, smoking cessation in special populations?)
   2. Were you able to implement tobacco trainings in your new employee orientations and annual training requirements? Why or why not?
2. Tell me about your overall experience with the TTTF project; for example, what services did you take advantage of?
   1. Trainings? Trainings and technical support from TTTF personnel? Policy creation support? Dissemination materials? Step-by-step toolkit? Provision of NRT? Providing group smoking counseling? Website best practices?
   2. What did you find most helpful?
3. What was your experience of implementing the TTTF program?
   1. Which program components did you successfully implement?
      1. Adoption of a tobacco-free policy? Can you tell me a bit more about your current tobacco-free policy? How is it different to the policy you had before you started this project, if any?
      2. Regular assessment and documentation of tobacco use among clients?
   2. Which program components did you find challenging and impractical to implement and why? Do you plan / want to implement them in the future? What would you need to do that?
4. What modifications did you make to the program to better suit the individual needs of your clients/center?
5. What was most surprising to you about the process of becoming tobacco-free?
   1. What did you learn in this process?
6. How has the process of becoming tobacco-free changed your center, either positively or negatively?
   1. Have you noticed any change in your organization’s culture? Have you staff supported this change?
   2. Changes in staff and/or clients or how they interact?
   3. Do you have any staff who smoke? Did any of them quit or made a quit attempt during the program? What was their experience?
7. What have been the main challenges that you faced in to implementing your agency’s tobacco-free workplace policy, and how did you, or what is still needed to overcome these barriers? (If needed, prompt: On the level of organizational resources, clinicians, clients?)
   1. Did your clients overall support it or not? What was their reactions? Did you have any problems with them not following the policies?
   2. Do you provide any tobacco cessation services to you clients now? Have they been willing to use these services? Were they overall willing to make a quit attempt or not interested? What kind of services were mostly used (counselling, NRT)? What difficulties you and your clients had when providing/receiving these services?
8. What challenges do you think you will face in sustaining your tobacco free workplace, if any?
   1. How will you/your center go about addressing them?
   2. Who will be the key players?
   3. What needs can you identify that would ease this process for you?
9. How would you describe the support you received from your agency’s leaders in implementing the TTTF program?
10. Part of this project involved collecting anonymous data on clients’ attempts at quitting smoking – how many made attempts to quit, how many successfully quit, etc. What has the process of collecting client data been like for you?
    1. Did you manage to collect client quit data? Why or why not?
    2. What do you need to facilitate this data collection process?
    3. What best practices can you share on how to improve/streamline this process?
11. What have been the main facilitators to delivering and sustaining your agency’s tobacco-free workplace policy? How do you think these facilitators can be enhanced/ supported? (If needed, prompt: On the level of organizational resources, clinicians, clients?)
12. Tell me about any “best practices” you developed in implementing the program that you might like to share with others:
    1. Practical tips?
    2. Development, or streamlining, of procedures?
    3. Was there anything that did not work as planned, anything that you wish you would have done differently?
13. What additional efforts would you like to see happening in your center to sustain and even enhance the tobacco free environment for your employees and clients?
    1. Potential examples: more training or “booster sessions”; forums to further discuss challenges and potential solutions; practical changes like identifying a “tobacco champion/coordinator” or implementing tobacco assessments systematically in electronic medical records, etc.
    2. What recommendations might you have for future expansions of the project?
14. We want to understand any concerns or challenges you might have faced in going tobacco-free. What would you most like us to know so that we can more fully understand your perspective on treating tobacco dependence among your clients and what challenges, reservations, or benefits you have encountered in implementing the TTTF program?
15. What else, if anything, you would like to share that we haven’t addressed?
